# Genetic analysis of phenotypic plasticity identifies *BBX6* as the candidate gene for maize adaptation to temperate regions

**DOI:** 10.3389/fpls.2023.1280331

**Published:** 2023-10-30

**Authors:** Yuting Ma, Wenyan Yang, Hongwei Zhang, Pingxi Wang, Qian Liu, Fenghai Li, Wanli Du

**Affiliations:** ^1^ College of Agronomy, Shenyang Agricultural University, Shenyang, Liaoning, China; ^2^ State Key Laboratory of Crop Gene Resources and Breeding, Institute of Crop Sciences, Chinese Academy of Agricultural Sciences, Beijing, China

**Keywords:** maize, plasticity, candidate gene, domestication, selective sweep

## Abstract

**Introduction:**

Climate changes pose a significant threat to crop adaptation and production. Dissecting the genetic basis of phenotypic plasticity and uncovering the responsiveness of regulatory genes to environmental factors can significantly contribute to the improvement of climate- resilience in crops.

**Methods:**

We established a BC1F3:4 population using the elite inbred lines Zheng58 and PH4CV and evaluated plant height (PH) across four environments characterized by substantial variations in environmental factors. Then, we quantified the correlation between the environmental mean of PH (the mean performance in each environment) and the environmental parameters within a specific growth window. Furthermore, we performed GWAS analysis of phenotypic plasticity, and identified QTLs and candidate gene that respond to key environment index. After that, we constructed the coexpression network involving the candidate gene, and performed selective sweep analysis of the candidate gene.

**Results:**

We found that the environmental parameters demonstrated substantial variation across the environments, and genotype by environment interaction contributed to the variations of PH. Then, we identified PTT(35-48) (PTT is the abbreviation for photothermal units), the mean PTT from 35 to 48 days after planting, as the pivotal environmental index that closely correlated with environmental mean of PH. Leveraging the slopes of the response of PH to both the environmental mean and PTT(35-48), we successfully pinpointed QTLs for phenotypic plasticity on chromosomes 1 and 2. Notably, the PH4CV genotypes at these two QTLs exhibited positive contributions to phenotypic plasticity. Furthermore, our analysis demonstrated a direct correlation between the additive effects of each QTL and PTT(35-48). By analyzing transcriptome data of the parental lines in two environments, we found that the 1009 genes responding to PTT(35-48) were enriched in the biological processes related to environmental sensitivity. *BBX6* was the prime candidate gene among the 13 genes in the two QTL regions. The coexpression network of *BBX6* contained other genes related to flowering time and photoperiod sensitivity. Our investigation, including selective sweep analysis and genetic differentiation analysis, suggested that *BBX6* underwent selection during maize domestication.

**Discussion:**

Th is research substantially advances our understanding of critical environmental factors influencing maize adaptation while simultaneously provides an invaluable gene resource for the development of climate-resilient maize hybrid varieties.

## 1 Introduction

Unforeseen climate changes, coupled with extensive climatic diversity, present significant challenges to crop production (Swarts et al., 2017). As stationary organisms, crop plants must adapt to these climate shifts through internal and phenotypic adjustments to ensure survival. The capacity of crops to adapt to diverse planting regions can be reflected in their phenotypic plasticity (Sultan, 2000). Phenotypic plasticity reflects the phenotypic variations across environments, and can be calculated by fitting regression models, in which phenotypic data in each environment are regressed against environmental factors (Arnold et al., 2019). Crop breeders aim to reduce phenotypic plasticity to confer stable performance across different planting regions. A comprehensive analysis of environmental parameters in distinct ecological regions serves as the foundation for investigating phenotypic plasticity and devising actionable strategies for developing climate-resilient crop varieties (Lobell et al., 2008).

Genetic analysis of phenotypic plasticity holds the potential to enhance crop improvement in the face of fluctuating environmental conditions. Earlier studies have unveiled both the similarities and disparities in the genetic architectures governing trait performance and plasticity in crops (Li et al., 2019a) indicating that enhancing trait performance and plasticity can be achieved with a certain degree of independence, and that it is possible design crop varieties with stable and desired field performance. Furthermore, genetic analysis of trait performance and plasticity paves the way for identifying genes linked to plasticity that can inform genome-guided breeding efforts, and for optimizing crop performance under favorable environments while mitigating yield losses during adverse climate changes.

Maize (*Zea mays ssp. mays*) was domesticated from its wild ancestor, teosinte (*Zea mays ssp. Parviglumis*), around 6,000 to 9,000 years ago in Mexico (Matsuoka et al., 2002). It was subsequently introduced to the southwestern regions of America approximately 4,000 years ago. The divergence of temperate maize from its tropical counterpart occurred after this period (Li et al., 2018). Human ancestors in the Americas selected maize based on agronomic traits, leading to its adaptation to varying climate conditions and indirect changes in its genome and transcriptome (Camus-Kulandaivelu et al., 2006). The knowledge-driven maize breeding efforts from the 19th century onwards further augmented its global adaptability, resulting in an eight-fold increase in seed production (Schnable and Springer, 2013). Nevertheless, our understanding of the intricate interplay between maize and its environment in adapting to diverse conditions remains limited.

B-box transcription factors (BBXs) are well-known in regulating flowering, photoperiod sensitivity, photomorphogenesis, and stress tolerance (Talar and Kiełbowicz-Matuk, 2021). The *Arabidopsis CO*, also known as *AtBBX1*, is the central regulator of photoperiod sensitivity (Valverde, 2011). *Hd1*, the rice homolog of *CO*, promotes flowering under short day conditions (Kojima et al., 2002), and is involved in the control of plant height (PH) and grain yield (Zhang et al., 2012). Moreover, several other *BBX* genes were associated with photoperiod sensitivity in *Arabidopsis* and rice (Talar and Kiełbowicz-Matuk, 2021). The maize homolog of *CO*, Conz1, controls photoperiod sensitivity by activating the FT-like gene ZCN8 (Meng et al., 2011; Wu et al., 2023). According to our literature review, no other BBX genes were involved in environmental sensitivity in maize except for *Conz1*.

Three genes positively regulate *ZCN8* expression, including *Conz1*, *ZmMADS1* (a MADS-box transcription factor), and *ID1* (a zinc finger protein transcription factor specific to monocotyledonous plants) (Lazakis et al., 2011; Meng et al., 2011; Guo et al., 2018). In contrast, *ZmRap2.7* (an AP2 family transcription factor) and *ZmCCT9* negatively regulate the expression of *ZCN8* (Salvi et al., 2007; Huang et al., 2018; Liang et al., 2019). *ZmMADS69* negatively regulates *ZmRap2.7* expression to relieve its repression of *ZCN8*, and leads to early flowering (Liang et al., 2019). *ZCN8* and *DLF1* (a bZIP transcription factor) interact to mediate the differentiation and formation of floral organ in the shoot apical meristem. *DLF* is homologous to *FD* gene in *Arabidopsis*, and its loss-of function can delay flowering time (Muszynski et al., 2006). ChIP-seq (Chromatin Immunoprecipitation sequencing) and RNA-seq analysis found that *DLF1* activated the expression of two MADS-box genes, *ZmMADS4* and *ZmMADS67*, by binding to their promoters. Knocking-out of *ZmMADS4* and *ZmMADS67* delays flowering time and increases leaf number (Sun et al., 2020). Above all, the pathway of *ZmMADS69*-*ZmRap2.7*-*ZCN8*-*DLF1*-*ZmMADS4*/*ZmMADS67* was known to regulate the photoperiod sensitivity and adaptation of maize. However, given the unpredictability of climate changes, there is a pressing need to uncover additional genes associated with maize adaptation, especially in elite lines used in hybrid maize breeding.

However, prior genetic and molecular studies on crop adaptation have primarily utilized flowering time as an indicator of phenotypic plasticity (Guo et al., 2020). It is essential to explore the adaptation of other traits to varying environments, particularly PH. PH is a pivotal trait linked to biomass and has implications for both yield and lodging resistance (Wang et al., 2023). Investigating the genetic foundation of PH and its plasticity holds significance for developing silage corn, requiring elevated biomass and PH (Wu et al., 2020), as well as high-density tolerant maize, necessitating shorter stature (Wang et al., 2023). Remarkably, few studies have focused on maize PH plasticity.

To dissect the genetic underpinnings of PH plasticity and identify candidate genes, we established a BC_1_F_3:4_ population utilizing elite inbred lines Zheng58 and PH4CV. We evaluated PH of this population across four distinct environments characterized by variations in environmental parameters. By exhaustive searching of the mean environmental parameters within a growth window, we identified an environment index closely correlated with the environmental mean. The reaction norm of PH to an environment index was as well modelled as that of PH to the environmental mean. By utilizing the slopes of reaction norms to represent PH plasticity, we identified two potential QTLs responsive to the environment index through a genetic analysis of PH plasticity. Transcriptome analysis unveiled a candidate gene, *BBX6*, that responded to environmental parameters. Selective sweep analysis indicated that *BBX6* underwent selection during the transition from teosinte to landrace. Our findings furnish QTLs and candidate genes for enhancing maize adaptation.

## 2 Materials and methods

### 2.1 Phenotypic and genotypic data

The BC_1_F_3:4_ population comprising 481 families has been detailed in a previous study (Li et al., 2019b). In summary, Zheng58 and PH4CV were used as the donor and recurrent parents respectively to generate 481 BC_1_F_3_ plants, which were subsequently self-pollinated to yield 481 BC_1_F_3:4_ families. It’s noteworthy that Zheng58 serves as the maternal parent of Zhengdan958, while PH4CV is the paternal parent of Xianyu335. Notably, Zhengdan958 and Xianyu335 stand as renowned hybrid varieties in China. PH of the BC_1_F_3:4_ families was assessed during the summers of 2016 and 2017 in Shunyi (BJ: Beijing) and Changji (XJ: Xinjiang), respectively. These four environments were denoted as 16BJ, 17BJ, 16XJ, and 17XJ (Li et al., 2019b). Pertinent geographic and environmental details of these four environments can be found in **Table S1**. For specifics on experimental design and field management, refer to the previously published description (Li et al., 2019b). PH was determined as the measurement from the ground to the tassel tip, with outliers removed based on 1.5 times the interquartile range. The PH values for all four environments are provided in **Table S2**.

Genotyping of the 481 BC1F3 plants was conducted by CapitalBio Corporation using a DNA array containing approximately 55,000 SNPs. Processing of the genotypic data yielded 11,781 polymorphic SNPs, and these data are publicly available (Li et al., 2019b).

### 2.2 Variance component analysis

Dissection of variance components was carried out in accordance with a previously reported methodology (Li et al., 2019b). In brief, the model can be expressed as:


PH=μ+G+E+G x E+Rep+ε,

where PH represents the PH of the i^th^ (i = 1, 2,…, 481) line within the m^th^ (m = 1, 2) replication in the k^th^ (k = 1, 2, 3, 4) environment. The terms denote the overall mean (μ), genotype effect (G), environment effect (E), genotype-by-environment interaction effect (G x E), replication effect nested within the k^th^ environment (Rep), and residual error (ε). Each effect was treated as a random effect, with a specific normal distribution. Heritability was calculated using the formula described previously (Li et al., 2019b).

### 2.3 Genomic selection analysis

Genomic selection was performed for single environments (GS_SE) and for integrating the G x E effect (GS_GE). In the case of GS_SE, the BGLR package (Pérez and de los Campos, 2014) was employed. The RKHS method was utilized to estimate parameters. The model can be represented as:


PH=Xβ+Kμ+ε,

where PH signifies the PH for each environment, β denotes the fixed effect, μ symbolizes the marker effect following a distribution of μ ~ N(0, K_σ_μ^^2^), X corresponds to the design matrix for the fixed effect, K stands for the n x n kernel matrix where n is the number of markers, and k represents the Gaussian kernel function. The RKHS model adopts a Bayesian framework for Gibbs sampling, employing model parameter settings of 10,000 iterations and a 2,000 burn-in.

The GS_GE model utilized the Multitrait function from the BGLR package, enabling Bayesian models with an arbitrary number of random effects to be fitted (Cheng et al., 2018). Phenotype in different environments were treated as distinct traits, leveraging the covariances of phenotype across different environments to predict trait values in specific environments (Burgueño et al., 2012; Pérez and de los Campos, 2014). Prediction was carried out with 200 five-fold cross-validations. The correlation coefficient between predicted and actual data was computed in each cross-validation, and prediction accuracy was the mean correlation coefficients across all cross-validations. The ggplot2 package was employed for visualization, and the ggpubr function was used to determin e whether the difference between GS_SE and GS_GE was statistically significant.

### 2.4 Searching the environmental index

Temperature and day length data was acquired from a public source as outlined by Guo et al. (Guo et al., 2020). Growing degree days (GDDs) follows the formular GDD = ((T_max_ + T_min_)/2 - T_base_), where T_max_ is the maximum temperature (°F), and is assigned as 100°F if T_max_ exceeds 100°F. T_min_ is the minimum temperature, and becomes 50°F if T_min_ is less than 50°F. T_base_ is the species-specific base temperature, and is 50°F for maize.Photothermal units (PTT) and photothermal ratio (PTR) follow the formulars: PTT = GDD x DL; PTR = GDD/DL.

To identify the critical environmental index, we employed the CERIS algorithm (github.com/jmyu/CERIS_JGRA) to establish the relationship between environmental parameters and environmental means. In the growth period before 62 days after planting (DAP), we computed the average of environmental parameters from the window of consecutive starting and ending days (DAP^i^ to DAP^j^), encompassing DL, GDD, PTT, and PTR. DAP^i^ and DAP^j^ represent the i^th^ and j^th^ DAP (i < j -5). For each environmental parameter, the mean parameter from DAP^i^ to DAP^j^ was computed, followed by the calculation of the correlation coefficient between the mean parameter and the environmental mean. The parameter–window combination that had the highest correlation coefficient with the environmental mean was employed as the environmental index.

### 2.5 GWAS analysis

The reaction norm captures the relationship between environmental mean/index and PH. The reaction norms of each genotype were obtained via regression of PH of each line against environmental mean/index (as explanatory variables). Two parameters, the slope and the intercept, were extracted from each reaction norm, with the slope representing PH plasticity.

The rrBLUP package (Endelman and Jannink, 2012) was utilized to perform GWAS analysis of the slopes derived from the two reaction norms. The intercept and average PH in each environment were employed as line performance for GWAS. P3D was set to false during GWAS execution. The threshold was determined by the Bonferroni correction, defined as -log_10_(0.1/Ne), where Ne signifies the number of effective markers, calculated via the SimpleM package (https://github.com/LTibbs/SimpleM). To assess the response of each QTL to the environmental index, the additive effect of each QTL in each environment were computed.

### 2.6 Transcriptome analysis

In July 2nd, 2018, the parental lines (Zheng58 and PH4CV) were planted in Haidian (Beijing). On August 9th, the decapitated internodes were sampled, with three replicates for each line. This data was utilized in our prior research (Zhou et al., 2022). Additionally, on May 16th, 2022, the same lines were planted in Langfang (Hebei), with decapitated internodes sampled on June 25th, also with three replicates for each line. RNA samples were extracted and subjected to RNA-seq library construction, following the method described previously (Zhou et al., 2022). The RNA-seq data generated in 2018 were published in our previous article (Zhou et al., 2022), while the RNA-seq data generated in 2022 are accessible on NCBI under BioProject number PRJNA1006801.

The RNA-seq data underwent filtration to remove low-quality reads, contaminants, and reads with N bases exceeding 5%. Hisat2 (Kim et al., 2015) was employed to align the clean reads to the B73_RefGen_V3 reference genome (www.maizegdb.org), and HTSeq tool (Anders et al., 2015) was used to quantify the number of uniquely mapped reads. The reading counts were normalized by library size, and the TMM normalization method was used to derive the CPM value for each gene in each sample (Robinson and Oshlack, 2010). Subsequently, the CPM values were normalized based on gene CDS length to obtain FPKM values (exon fragments per thousand bases per million reads). DESeqDataSetFromMatrix function of the R package DESeq2 was used to obtain the differentially expressed genes with the criteria of |log_2_(FoldChange)|>=1 and *P*_adj_ < 0.05.

Genes showing significant differential expression in PH4CV between the two environments, and showing non-significant differential expression in Zheng58 between the two environments were used for GO enrichment analysis, which was carried out by using the enrichGO function of the R package clusterProfiler. Significance was declared by an adjusted *P* value (*P*-adjust) below 0.05.

The three replications of the filtered RNA-seq data of each sample were combined to assemble the transcribed sequences following the command: Trinity –genome_guided_bam –genome_guided_max_intron 10000 –max_memory 150G –CPU 40 –output. The assembled coding sequence of Zheng58 and B73 were blasted against BBX6 coding sequence of B73, and the best matches of coding sequences of the two parental lines were considered as BBX6 coding sequences.

### 2.7 Construction of the coexpression network of *BBX6*

RNA-seq data were sampled from 31 tissues or stages of B73 (a classical maize inbred line), and two biological replicates were collected for each tissue or stage (Han et al., 2023). The procedures of RNA isolation, library construction and sequencing, and quantification of RNA were described previously (Han et al., 2023). Genes detected in more than 10 tissues or stages was used to construct a coexpression network using WGCNA (v.1.70-3) with default parameters, and genes showing strong correlation with *BBX6* were extracted from the coexpression network.

### 2.8 Selective sweep and genetic differentiation analysis

Given the reliance of variety adaptability on environmental shifts, we conducted a selective sweep analysis of *BBX6* to ascertain whether it was subjected to selection during domestication or improvement processes. This analysis utilized maize haplotype version 3 (HapMap 3), derived from whole genome sequencing data of maize, landraces, and teosinte lines (Bukowski et al., 2018). Based on B73 reference genome V3 (https://www.maizegdb.org/), we extracted the promoter, 5’ UTR, coding sequence, and 3’ UTR sequence of *BBX6* from three populations, encompassing 21 teosinte lines, 26 landraces, and 1486 inbred lines. Population genetic differentiation analysis (Fst) and nucleotide diversity analysis (Pi) were performed using VCFtools (Weir and Cockerham, 1984).

Sliding window calculation was chosen to enhance the sensitivity of selection signals (Ma et al., 2015). The parameter settings for calculating Fst values were –fst-window-size 100 bp and –fst-window-step 25 bp, while the parameter settings for calculating Pi values were –pi window-size 1000 bp and –pi-window-step 100 bp.

## 3 Results

### 3.1 Genotype by environment interaction contributes phenotypic variations

**Figure 1:**
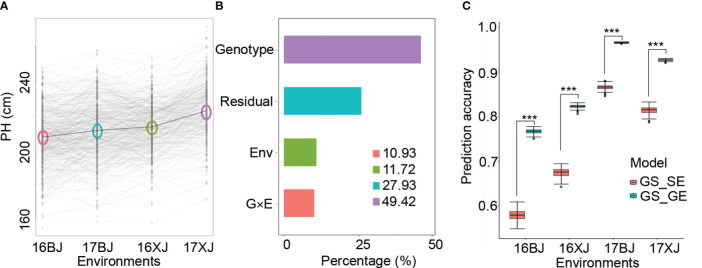
Genotype by environment interaction contribute to PH variation. **(A)** PH variations across environments; the circles in the plot indicate the environmental mean; **(B)** variances of different components; **(C)** comparison of the prediction accuracy of GS_SE and GS_GE models, and *** indicates P < 0.001 based on *t*-test analysis.

An initial examination of PH distributions revealed notable differences among different environments. PH was highest in the 17XJ environment, followed by 16XJ, 17BJ, and 16BJ (**Figure 1A**). Additionally, the performance of most lines displayed variations across different environments (**Figure 1A**). High correlation coefficients between replications within the same environment indicated that these variations were not due to errors (**Table S3**). Furthermore, the heritability of PH was substantial, reaching a value of 0.83. Therefore, the variations of PH (**Figure 1A**) could be attributed to genotype by environment interactions. Subsequent variance component analysis highlighted that genotypic variance was the primary component, but G x E variance also played a significant role in the variations of PH across environments (**Figure 1B**). Moreover, exploring whether GS_GE outperformed GS_SE models revealed that incorporating the G x E effect greatly increased the prediction accuracy of GS models (**Figure 1C**). This finding further supported the influence of G x E interactions on the variations of PH across different environments.

### 3.2 Variations of environmental parameter

**Figure 2:**
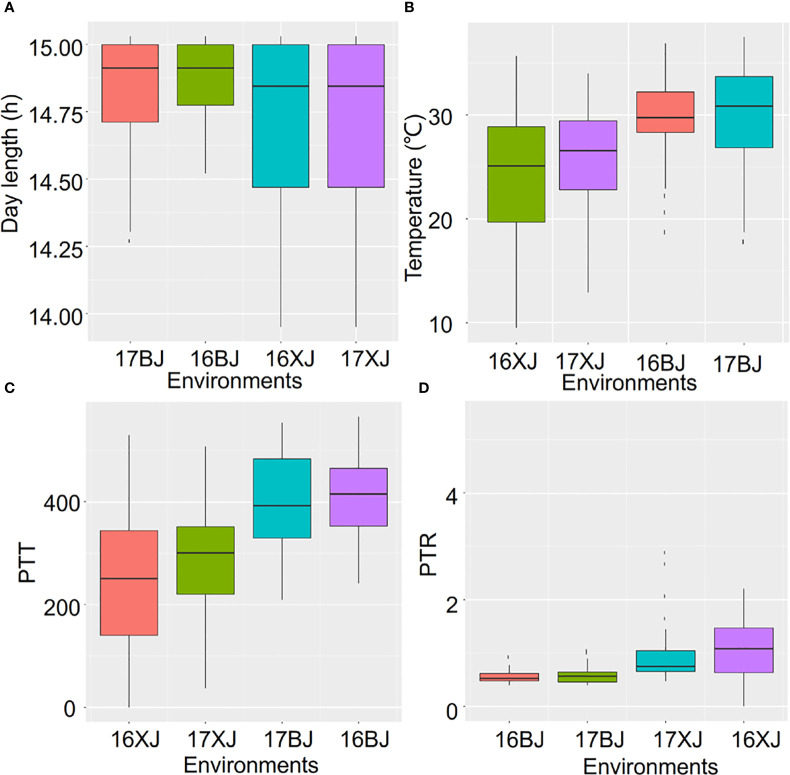
The variations of environment parameters across environments. The environment parameters contain day length **(A)**, Temperature **(B)**, PTT **(C)**, and PTR **(D)**.

In light of the above results, an investigation into the variations of environmental parameters across the four environments was conducted. These environments differed in terms of altitude, latitude, longitude, and planting date (**Table S1**). Although Beijing and Xinjiang shared similar latitudes, indicating potentially comparable day lengths, Xinjiang exhibited more pronounced variations in day length during both 2016 and 2017 compared to Beijing (**Figure 2A**). Notably, the altitude of Xinjiang exceeded that of Beijing (**Table S1**), which could account for the lower temperatures observed in Xinjiang (**Figure 2B**). Analyzing temperature changes across 2016 and 2017 showed that the summers of 2017 were hotter than those of 2016 in both locations (**Figure 2B**). This unexpected climate change underscored the variability of climate across years. The variations in temperature and day length led to corresponding variations in PTT and PTR across the four environments (**Figures 2C, D**). Such variations in environmental parameters, both across years and locations, further complicate the impact of the environment on maize performance.

### 3.3 Searching for an environmental index closely correlated with PH

**Figure 3:**
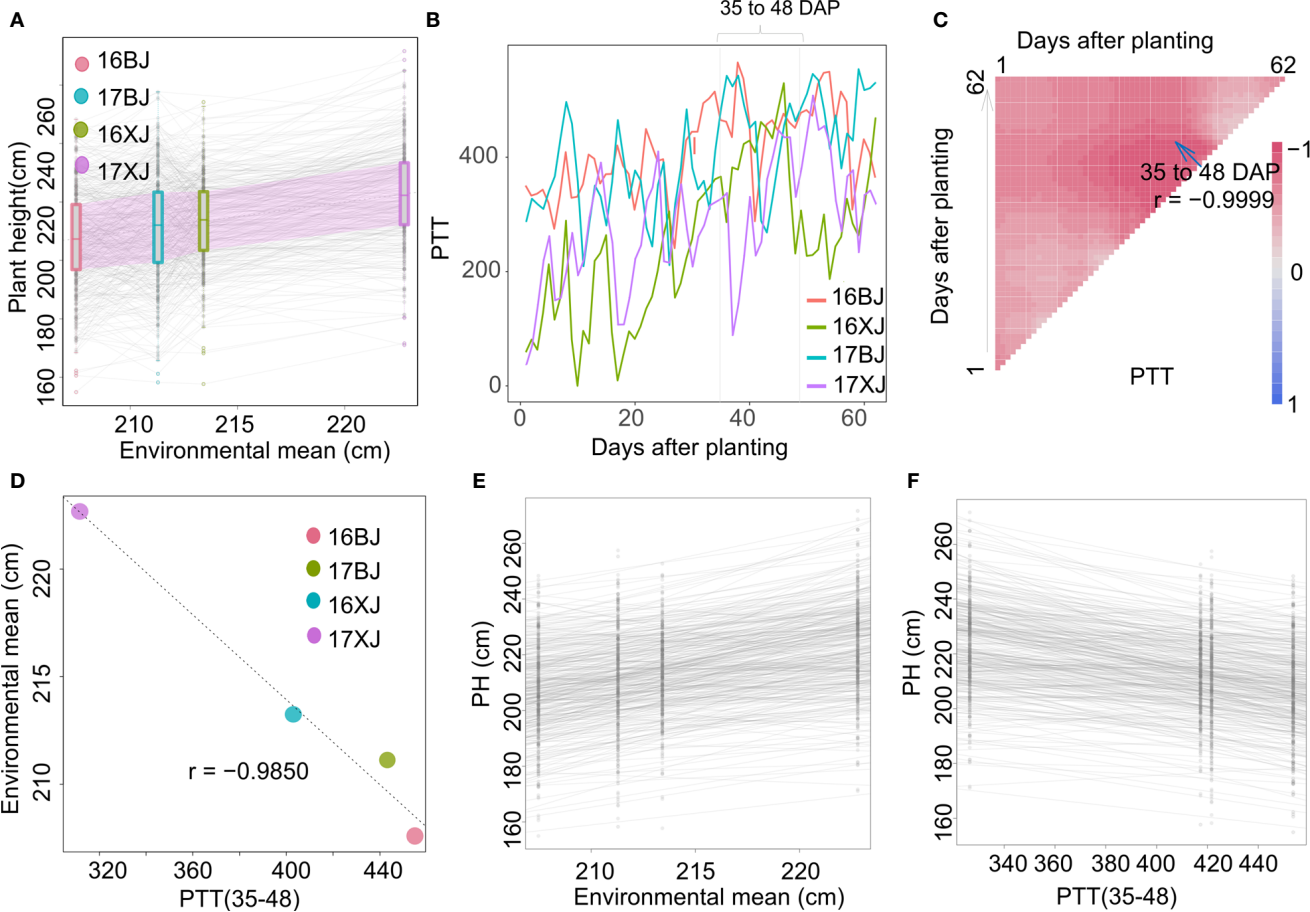
Identifying an environmental index using environmental and performance data. **(A)** PH of most lines generally increase with the increase of the environmental mean; **(B)** PTT variations from 1 to 62 DAP in four environments; **(C)** search for the most indicative growth window within which mean PTT has the strongest correlation with the environmental mean; **(D)** the correlation between mean PTT(35-48) and environmental mean; **(E)** the reaction norm using environmental mean as the explanatory variable; **(F)** the reaction norm using mean PTT(35-48) as the explanatory variable.

PH is heavily influenced by environmental factors, particularly temperature and day length (Mu et al., 2022). Although the population was evaluated in only two locations, PH of most lines generally have a gradual increase along with the increase of the environmental mean (**Figure 3A**), indicating environment factors play a complicate role in determining PH performance across environments. In-depth analysis of the exact influence of environmental factors on PH was undertaken by examining the reaction of PH to average temperature, day length, GDD, and PTR. However, PH exhibited patterns that were not well-modeled by these individual factors (**Figure S1**). Given this complexity, an effort was made to identify an environmental index that could serve as a surrogate for environmental mean. The approach involved averaging the environmental parameters within specific growth windows using the CERIS algorithm (Li et al., 2021). Notably, the oscillating increase in PTT values was observed across the four environments (**Figure 3B**), and the average value of PTT from the window of 35 to 48 DAP, labeled as PTT(35-48), displayed a stronger correlation with the environmental mean (**Figure 3C**) than the average values of other parameters within various growth windows (**Figures S2A–C**). Because there is overlapping among windows, the mean PTT values of the windows around 35-48 DAP also displayed strong correlations with the environmental mean (**Figure 3C**). In the growth period from 35 to 48 DAP, substantial differences were evident among the environments, particularly from 35 to 40 DAP (**Figure 3B**). Correspondingly, the mean PTT(35-48) values exhibited strong correlations with the environmental mean (**Figure 3D**). By establishing the relationship between PH and PTT, the critical growth stage influencing PH was narrowed down to 35 to 48 DAP – coinciding with the maize stem-elongation stage. Reaction norms based on the environmental mean and PTT(35-48) as explanatory variables were well-modeled (**Figures 3E, F**), with the two slopes exhibiting a highly strong correlation (r = -0.97). The negative correlation is caused by the negative correlation between environmental mean and PTT(35-48) (**Figure 3D**).

### 3.4 Genetic basis of phenotypic plasticity

**Figure 4:**
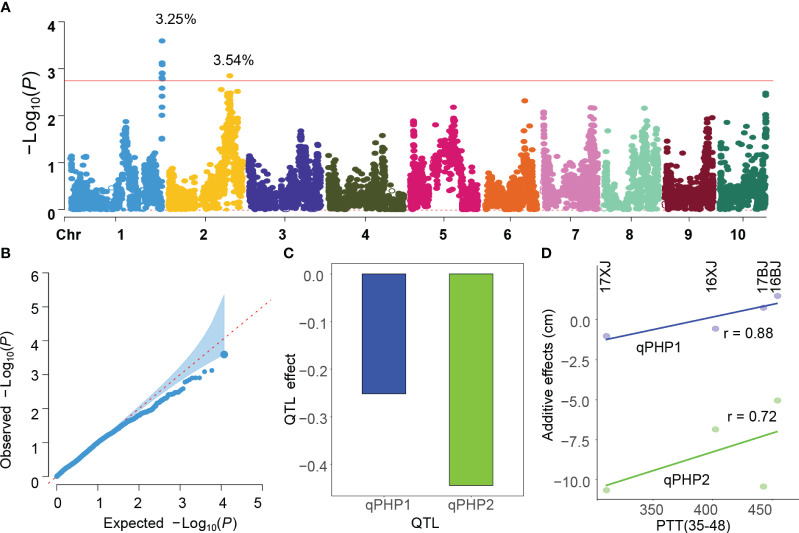
GWAS analysis of PH plasticity. **(A, B)** and B are Manhattan and QQ plot obtained by performing GWAS using the slope obtained from **Figure 3E** as the phenotype, and phenotypic variances explained by the QTLs are added in the Manhattan plot; **(C)** is the additive effects of the two QTLs on chromosome 1 and 2, and negative values indicate PH4CV genotypes have positive contributions; **(D)** the correlation coefficients (r) between PPT(35-48) and additive effects.

GWAS utilizing the two slopes extracted from the reaction norms as phenotypes revealed the identification of two consistent QTLs on chromosomes 1 and 2 (**Figures 4A**, **S3**). These QTLs were denoted as qPHP1 and qPHP2 (PHP indicating PH plasticity). The peak positions of qPHP1 and qPHP2 are 300,034,330 bp and 196,386,747 bp, respectively. The QQ plot illustrated that the population structure was effectively controlled (**Figure 4B**). The proportion of phenotypic variance explained (PVE) for qPHP1 and qPHP2 was 3.25% and 3.54%, respectively, with qPHP2 (-0.44) possessing a larger additive effect compared to qPHP1 (-0.25) (**Figure 4C**). Interestingly, the PH4CV genotypes at the two QTLs have positive contributions to phenotypic plasticity, indicating PH4CV genotypes are more sensitive to environmental changes than the Zheng58 genotypes.

Given the pivotal role of the 35-48 DAP period in maize growth (**Figures 3B, C**), it was hypothesized that the detected QTLs’ additive effects might respond to PTT(35-48) variations. To prove this hypothesis, we performed GWAS analysis using the PH in each environment, and the intercept of the reaction norm that regresses PH against the environmental mean.

This analysis unveiled that the absolute values of additive effects of both QTLs decreased with the increase of PTT(35-48) (**Figure 4D**), which mirrors the negative correlation between PTT(35-48) and PH (**Figure 3D**). Additionally, GWAS demonstrated that qPHP2 consistently influenced PH across environments, whereas qPHP1 exhibited no association with PH in any environment (**Figure S4**). This indicated both overlapping and divergence between the genetic basis of trait performance and phenotypic plasticity.

### 3.5 RNA-seq analysis indicates that BBX6 is the candidate gene in qPHP2 region

**Figure 5:**
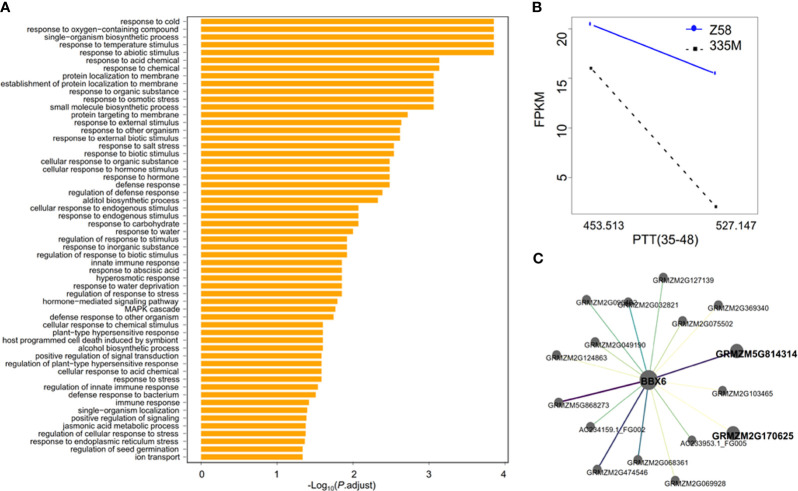
RNA-seq analysis reveals that BBX6 is the candidate gene underlying qPHP2 **(A)** GO enrichment analysis of the 1009 genes responding to PTT(35-48); **(B)** Responsive expression of BBX6 to PPT(35-48); **(C)** BBX6 coexpression network. With the increase of weight, the colors of edges changed from yellow to purple, and the width of edges become thicker.

Focusing on the critical 35-48 DAP growth period, RNA samples were extracted from elongating internodes at th is period. Given PH4CV’s stronger response to PTT(35-48) variations, a gene search conducted to identify differentially expressed genes with significant responses to PTT(35-48) in PH4CV and non-significant responses in Zheng58 yields 1009 genes (**Table S4**). GO annotation analysis found that these genes are enriched in multiple biological processes such as responsive to temperature and abiotic stress (**Figure 5A**), indicating that most of these genes are sensitive to environmental fluctuation. Five and eight genes were respectively located in the qPHP1 and qPHP2 regions (**Table S4**), among which *BBX6* (spanning from 195,818,312 to 195,828,932 bp on chromosome 2) was very close to the peak position of qPHP2, and was identified as the candidate gene within qPHP2. The homolog of *BBX6* functions in rice and *Arabidopsis* was associated with PH and flowering time (Wang et al., 2014; Chai et al., 2021; Cao et al., 2023), further supporting *BBX6* as the candidate gene in the qPHP2 region.

The expression level of *BBX6* decreased with increasing PTT(35-48) in PH4CV (**Figure 5B**), mirroring the sensitivity of the PH4CV genotype of qPHP2 to PTT(35-48) changes (**Figure 4D**). However, there were no sequence variants in the coding sequences of BBX6 of the two parental lines, and only a SNP in the 3’ UTR regions were detected (**Figure S5**). To prove that BBX6 is the candidate gene, the coexpression network of *BBX6* was extracted from the coexpression network constructed using the transcriptomic data of 31 tissues or stages (Han et al., 2023). There are 17 genes in the coexpression network (**Figure 5C**). Among these genes, the *Arabidopsis* homologs of *GRMZM5G814314* and *GRMZM2G170625* are respectively *UBC1* (*AT1G14400*) and *JAL1* (*AT3G16470*), which were associated with flowering time (Xu et al., 2009; Xiao et al., 2015). Especially, *UBC1* is involved in activating *FLC* expression and repressing flowering (Xiao et al., 2015) and *JAC1* Influences RNA processing of *FLC* antisense transcripts in *Arabidopsis* (Xu et al., 2009). *FLC* is key to photoperiod and vernalization perception and antagonistically regulates *FT* to influence the flowering time of plants (Searle et al., 2006). Therefore, *BBX6* may control PH plasticity by regulating photoperiod sensitivity.

### 3.6 Selective sweep analysis and genetic differentiation analysis revealed that BBX6 was selected during maize domestication

**Figure 6:**
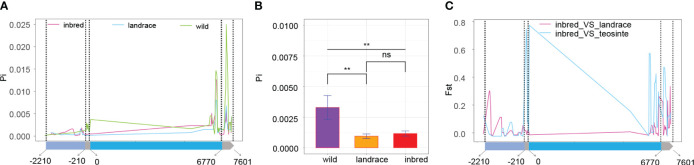
Selective sweep analysis of BBX6 **(A)** the Pi values of the three populations. The gene structure is demonstrated at the bottom. The four regions from left to right are promoter (2000bp), 5’UTA, coding sequence, and 3’UTA; **(B)** Significant test of Pi values among the three populations; ** and ns indicate P < 0.01 and P > 0.05, respectively. **(C)** Fst along the gene sequence of BBX6.

Subsequent exploration focused on whether *BBX6* underwent selection during domestication or improvement. The Pi ratio of teosinte exceeded that of landrace and inbred lines, with differences between landrace and inbred lines proving non-significant (**Figures 6A, B**), particularly in coding sequence and 3’ UTR regions. These results suggested that *BBX6* underwent selection during domestication. Evaluating genetic divergence between populations using *BBX6* genotypic data indicated greater divergence between maize and teosinte than between maize and landrace (**Figure 6C**). This observation further supported the notion that *BBX6* was selected during maize’s divergence from teosinte.

## 4 Discussion

The current era witnesses ongoing climate changes and increasing global temperatures, with potential threats posed to crop production (Keith, 2021). The development of climate-resilient crops exhibiting low plasticity across different environments is of paramount importance. However, our knowledge of the genetic and molecular mechanisms governing phenotypic plasticity remain limited. This study’s comprehensive approach, encompassing the establishment of a large population derived from elite inbred lines in China, field performance evaluations across diverse environments, assessing the variations of environmental parameters, identification of the key environmental index, integration of genetic and transcriptomic analyses, and genetic analysis of the candidate gene, offers valuable insights into maize plasticity.

Field performance emerges from the interplay of genotypic and environmental interactions (Li et al., 2022). The current findings substantiate that G x E variance significantly contributes to phenotypic variations. The significance of G x E interactions in influencing field performance is reinforced by the enhanced prediction accuracy of GS models upon incorporating the G x E effect. These results underscore the pivotal role of G x E interactions in shaping field performance across different environments, and is the basis for the searching of critical environment index in the next step. By demonstrating environmental parameters in the growth period (before 62 DAP) across environments, this study sheds light on the unique aspects of temperature, day length, GDD, and PTR variations across environments. Especially, we witness a great temperature increase in 2017, which is due to unexpected climate changes. This complexity of environmental variations adds an additional layer of challenge to the already intricate interplay between genetics and environment in shaping plant performance.

Identification of an environmental index closely correlated with performance offers insights into understanding the precise influences of environmental factors on maize growth. By investigating the relationship between environmental mean and environment parameters with varying growth window, we found that 35-48 DAP is the critical period determining PH, and PTT(35-48) can be used as a reliable surrogate for environmental influence (**Figures 3B, C**). Identification of the two QTLs for PH plasticity support the reliability of PTT(35-48) for surrogating the overall environmental factors (**Figures 4A**; **S3**). Importantly, using PTT(35-48) to surrogate environmental influences allow us to investigate the relationship between additive effects and PTT(35-48) variations, which underscores the interplay between genetics and environment in shaping phenotypic plasticity. Specifically, the additive effects of qPHP2 increased with the increase of PTT(35-48) (**Figure 4D**). Moreover, the additive effects of qPHP1 are nearly zero in Beijing, and the genotype of PH4CV in qPHP1 increase PH only in Xinjiang (**Figure 4D**). Interestingly, by performing GWAS analysis of the intercept, we find that qPHP2 is associated with the intercept and qPHP1 is not, indicating that qPHP1 and qPHP2 have divergent effects across environments, and qPHP2 had consistent effect on both intercept and slope.

Using diversity panels of maize wheat and oat, Li et al. found that QTL controlling the intercept and slope of flowering time showed great differences in their locations and effects (Li et al., 2021). In another research based on flowering time of a rice biparental population, Guo et al. found that the QTL controlling the intercept and slope were in the same genomic regions, but had different phenotypic contributions (Guo et al., 2020). These conclusions, together with our findings, highlight the nuanced interplay and complicated mechanism between trait performance and plasticity.

In plants, plant growth can be influenced by photoperiod and temperature (Song et al., 2013; Osnato et al., 2021). In this study, PTT is calculated by day length and growing degree days, and is related to photoperiod and temperature. Therefore, photoperiod and/or temperature are related to the control of PH plasticity of the tested population. In rice, *Ghd8* interacts with *Ghd7* to controls rice photoperiod sensitivity, and both genes affect heading date, PH and grain yield of rice (Wang et al., 2019). *Ghd7* is a central regulator of plant growth and development. Temperature regulates *Ghd7* expression, which is quantitively associated with field performance (Weng et al., 2014). Maize genes such as *ZmCCT* and gigantea was involved in photoperiodic control of plant growth (Bendix et al., 2013; Yang et al., 2013). Based on these clues, we suspect that photoperiod and/or temperature regulate *BBX6* expression, and the differences of photoperiod/temperature among the four environments caused the differences in *BBX6* expression, leading to different additive effects of PH4CV genotype at the qPHP2 locus.

Owning to its differential expression in response to PTT(35-48) and the coexpression relationship with genes potentially associated with flowering time and photoperiod sensivity (**Figures 5B, C**), *BBX6* was selected as the candidate gene for phenotype plasticity. Moreover, the BBX gene family were known to be involved in photoperiod sensitivity and adaptation. Especially, *Arabidopsis CO* gene and its homologs in maize, rice, and sorghum are well known as a photoperiod regulated activator of flowering. Because CO belongs to a large family, and members in this family have diverse functions (Gangappa and Botto, 2014), we further retrieve the closest homologs of maize *BBX6* by protein-to-protein BLAST. The *Arabidopsis* genes showing the highest similarity to maize *BBX6* is *BBX19*, followed by *BBX18*. *BBX19* interacts with *CO* to repress FT transcription, and constitutive expression of BBX19 delays flowering and decreases PH under inductive photoperiods (Wang 2014). Both BBX19 and BBX18 have crucial roles in fine-tuning circadian rhythm (Yuan et al., 2021). As the closest homolog of BBX19 and BBX18, *BBX6* might regulate PH by responding to photoperiod variations across different environments.

The breeding history was divided into Breeding 1.0 to Breeding 4.0 (Wallace et al., 2018). Breeding 1.0 indicated domestication of wild plants by ancient farmers. Breeding 2.0 used statistics and experimental design to assist the selection of desired plants. Breeding 3.0 combined genotypic and phenotypic data to improve breeding efficiency. We are now at the beginning of Breeding 4.0 stage with the help of multiple cutting-edge technologies. Crop domestication corresponded to Breeding 1.0 stages, and crop improvement started from Breeding 2.0. Both crop domestication and crop improvement changed agronomic traits and indirectly changed the sequences of genes associated with crop performance. Investigating the selection signature and genetic divergence of these genes increased our knowledge about what happed during the Breeding 1.0 stage, after the Breeding 2.0 stage, and perhaps would be advisable for crop improvement in the Breeding 4.0 stage. Through performing selective sweep analysis, we found that *BBX6* was selected during maize domestication, and the coding sequencing underwent strong selection. Genetic differentiation analysis further supported the result of selective sweep analysis. The gene expression analysis, gene coexpression analysis, and selective sweep analysis of *BBX6* indicated that *BBX6* could be a potential target gene for improving maize adaptation.

## Data availability statement

The data presented in the study are deposited in the NCBI repository, accession number PRJNA1006801.

## Author contributions

HZ: Conceptualization, Funding acquisition, Writing – review & editing. YM: Formal Analysis, Investigation, Writing – original draft. WY: Data curation, Formal Analysis, Investigation, Writing – original draft. PW: Investigation, Writing – original draft. QL: Investigation, Writing – original draft. FL: Writing – review & editing. WD: Conceptualization, Funding acquisition, Writing – review & editing.

## Supplementary material

Supplementary Figure 1 Reaction norms using mean GDD, day length, PTT, and PTR as the explanatory variables

Supplementary Figure 2 The correlation coefficients between environmental mean and the mean environment parameters within a growth window Note: Mean day length from 1 to 53 DAY **(A)**, GDD from 38 to 48 DAP **(B)**, and PTR from 38 to 48 DAP **(C)** have the strongest correlation with environmental mean

Supplementary Figure 3 Manhattan plot for the slope obtained from

Supplementary Figure 4 Manhattan plot of GWAS using the intercept and mean PH as the phenotype

Supplementary Figure 5 Gene structure of BBX6 in Zheng58 and PH4CV Note: The three regions from left to right are 5’UTR, coding sequence, and 3’UTR.
