# Supplementary figures and images for: Genetic analysis of phenotypic plasticity identifies BBX6 as the candidate gene for maize adaptation to temperate regions
Source: Front Plant Sci. 2023 Oct 30;14:1280331. doi: 10.3389/fpls.2023.1280331 (PMC10642939; doi:10.3389/fpls.2023.1280331)

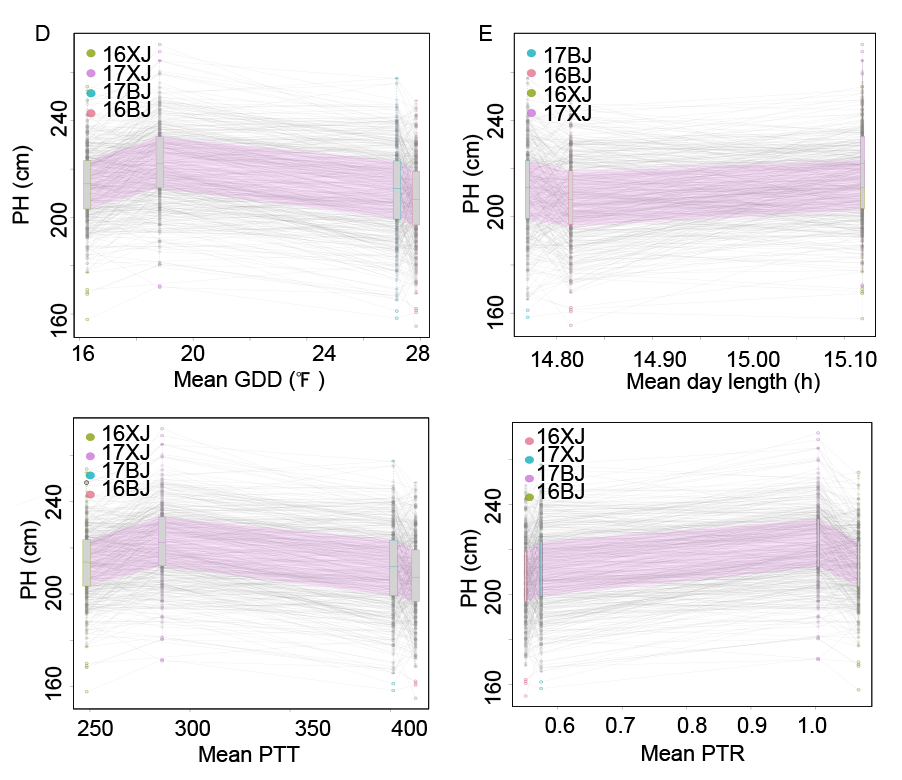

Supplement: Supplementary Figure 1 — Reaction norms using mean GDD, day length, PTT, and PTR as the explanatory variables [file Image_1.tif]

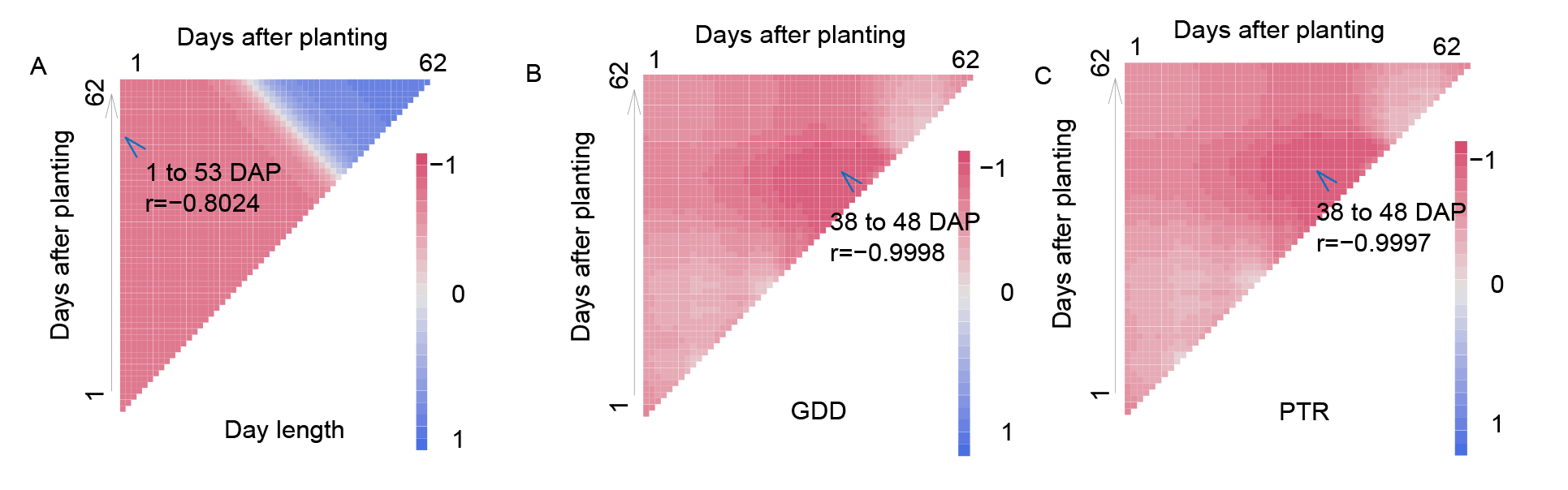

Supplement: Supplementary Figure 2 — The correlation coefficients between environmental mean and the mean environment parameters within a growth window Note: Mean day length from 1 to 53 DAY (A), GDD from 38 to 48 DAP (B), and PTR from 38 to 48 DAP (C) have the strongest correlation with environmental mean [file Image_2.tif]

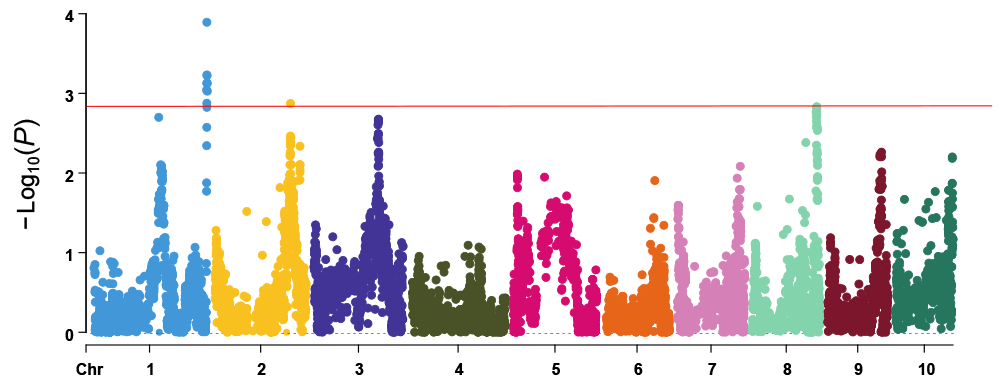

Supplement: Supplementary Figure 3 — Manhattan plot for the slope obtained from [file Image_3.tif]

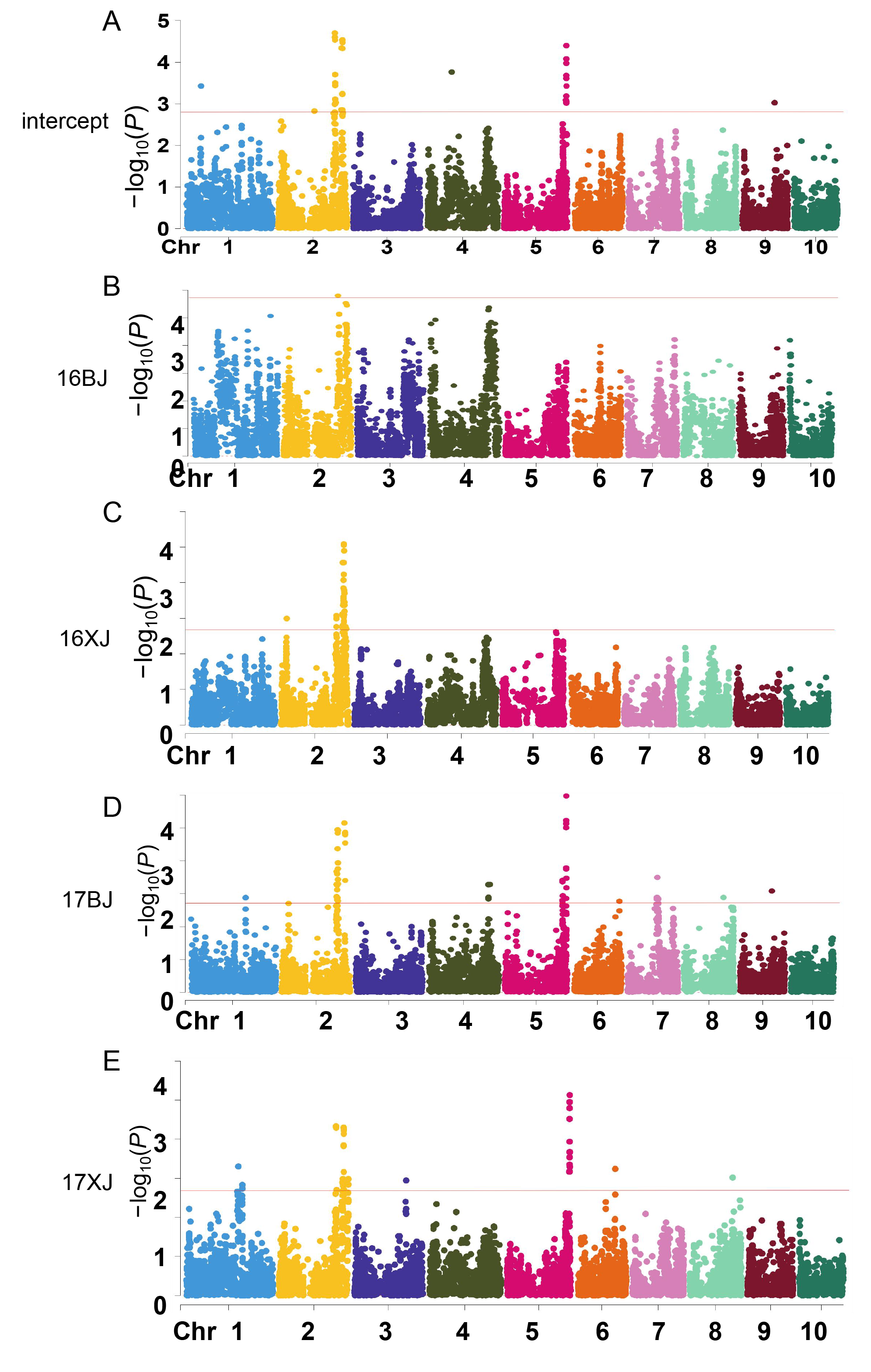

Supplement: Supplementary Figure 4 — Manhattan plot of GWAS using the intercept and mean PH as the phenotype [file Image_4.tif]

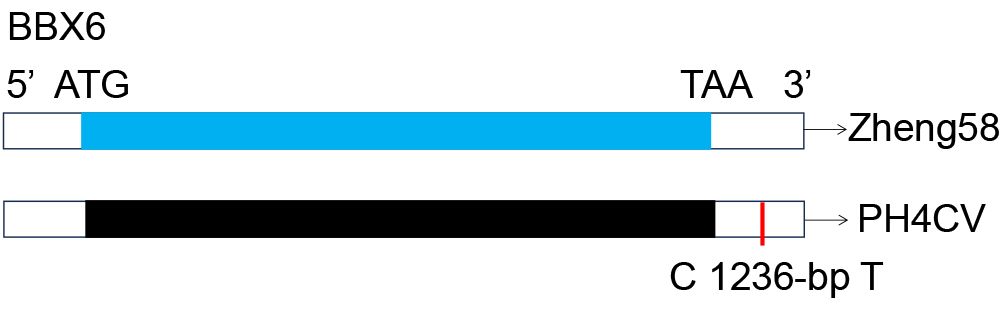

Supplement: Supplementary Figure 5 — Gene structure of BBX6 in Zheng58 and PH4CV Note: The three regions from left to right are 5’UTR, coding sequence, and 3’UTR. [file Image_5.tif]
